# Mapping terminology and domains related to spirituality in oncology: scoping review

**DOI:** 10.1007/s00520-026-11003-3

**Published:** 2026-07-24

**Authors:** Joao Pedro Santos Nascimento, Caique Mariano Pedroso, Alexandre Caroli Rocha, Marcio Ajudarte Lopes, Hercílio Martelli-Júnior, Horace M. DeLisser, Alan Roger Santos-Silva

**Affiliations:** 1 Department of Oral Diagnosis, Piracicaba Dental School, University of Campinas, Av. Limeira, Postal Code:13414-016, Piracicaba, São Paulo, 901 Brazil; 2 School of Medicine, NUPES - Research Center in Spirituality and Health, Federal University of Juiz de Fora (UFJF), Juiz de Fora, MG Brazil; 3 Oral Pathology and Oral Medicine, State University of Montes Claros, Montes Claros, Brazil; 4 Perelman School of Medicine, University of Pennsylvania, Philadelphia, USA

**Keywords:** Spirituality, Religion and medicine, Review, Neoplasms, Terminology as topic

## Abstract

**Purpose:**

To map and synthesize how spirituality is defined in the oncology literature, identifying the key domains associated with the concept.

**Methods:**

A scoping review was conducted following PRISMA-ScR guidance. PubMed, Scopus, Web of Science, Embase, and LILACS were searched, along with gray literature. Definitions and conceptual domains were synthesized using inductive thematic analysis, descriptive domain analysis, and a complementary content-analytic step.

**Results:**

A total of 104 studies were included in the review. Six core themes were identified: search for meaning and purpose, transcendence and connection to something greater, relational connectedness, inner peace and well-being, faith and belief in the sacred, and coping and existential resources. Definitions of spirituality varied widely, with earlier studies emphasizing religious frameworks and later ones adopting broader existential and psychosocial interpretations. Geographic trends highlighted cultural influences on the conceptualization of spirituality. The theme of meaning and purpose was the most consistently represented across geographic and temporal contexts.

**Conclusion:**

Spirituality in oncology is a multidimensional construct shaped by cultural, temporal, and contextual factors. Although the theme of search for meaning and purpose was represented across the largest number of definitions in the complementary content analysis, establishing a common definition of spirituality remains elusive. These findings underscore the importance of culturally sensitive approaches to spiritual care in oncology, recognizing how spirituality is conceptualized in relation to patient resilience and well-being.

**Supplementary Information:**

The online version contains supplementary material available at 10.1007/s00520-026-11003-3.

## Introduction

Cancer remains a leading cause of death worldwide. According to the latest estimation by the International Agency for Research on Cancer (IARC) of the World Health Organization, there have been approximately 20 million new cancer cases and nearly 10 million deaths related to the disease in the year of 2022 [1]. Although the 5-year survival rate of patients with cancer has improved in the last decades in response to the continuous improvement of medical technology, cancer is still a harmful disease that affects the patients physically and mentally [2]. The processes of diagnosis and treatment are often accompanied by pain, physical limitations, elevated levels of anxiety and depression, changes in self-image, and disruptions in social relationships, all of which considerably reduces quality of life [3]. Effective care extends beyond biomedical treatment and symptom management, requiring a comprehensive approach that also addresses the patient’s physical, emotional, social, and spiritual needs [4].

Spirituality has been recognized as an important dimension in supporting oncological treatment. It encompasses diverse cultural, religious, and existential perspectives, and has been linked to meaning, transcendence, coping, and relational connectedness [5]. In recent decades, evidence has increasingly demonstrated its association with resilience, quality of life, and even biological outcomes such as reduced inflammation and improved immune function [6, 7]. Spirituality is often regarded as synonymous with religion or religiosity, but there are considerable differences between these two dimensions [8]. While religiosity refers to adherence to specific religious practices, beliefs, and institutions, spirituality is a broader concept related to the search for meaning, purpose, and connection in diverse areas and aspects, which may or may not involve religious elements.

Despite its recognized importance, the literature lacks conceptual consensus regarding spirituality [6]. Terms such as "spiritual care," referring to interventions that address patients' spiritual needs [9], "existential support," which focuses on meaning and purpose during illness [10], and “spiritual well-being,” that describes a sense of peace and connection and is used as an outcome measure [11], are often used to describe different dimensions of the spiritual domain, yet their meanings are frequently blended under the broad label of spirituality. Communication is a cornerstone to healthcare as it shapes how clinical concepts are interpreted and applied [12]. In oncology, this is especially relevant, as terminology influences patients’ perceptions of illness, engagement in shared decision-making, and overall psychological well-being and treatment outcomes. For professionals, clear and standardized language enhances the interpretation of scientific evidence and supports the accurate translation of evidence-based practices into clinical care [13, 14].

While prior studies have examined spirituality in nursing, psychology, and general health contexts, oncology presents unique challenges. Cancer confronts patients with issues of mortality, suffering, and uncertainty, making spirituality a salient dimension of care [15–17]. Nevertheless, no comprehensive synthesis has previously focused on how spirituality is defined in oncology literature. To address this gap, we conducted a scoping review to map and synthesize definitions and conceptual domains associated with spirituality in oncology, guided by the research question: “How is spirituality conceptualized in oncology studies, and what are the key domains associated with it?”.

## Materials and methods

### Protocol and registration

This scoping review (ScR) was conducted in accordance with the Joanna Briggs Institute (JBI) Reviewer’s Manual for Scoping Reviews [18] and the Preferred Reporting Items for Systematic reviews and Meta-Analyses extension for ScR (PRISMA-ScR) checklist [19]. The review protocol was prospectively registered on Open Science Framework (https://osf.io/zwhuk).

### Eligibility criteria

The review was structured using the Population, Concept, and Context (PCC) framework, as follows: Population: oncology patients; Concept: definitions and domains related to spirituality; Context: oncology care and survivorship settings.

Eligible studies included patients diagnosed with cancer who were either undergoing active treatment, in the post-treatment phase, or in palliative care. We included qualitative studies and quantitative observational studies involving oncology patients that provide original or referenced definitions of "spirituality" or “spirituality in health”, and reference domains related to spirituality (e.g., religion, therapies, rituals, beliefs, or other practices).

Excluded from this scoping review were letters, reviews, short communications, personal opinions, conference abstracts, and laboratory research. Also excluded were studies that involved patients not undergoing cancer treatment, that failed to provide a definition of spirituality, whose full texts were not available or addressed topics beyond the focus of the review and/or were in languages other than English, Spanish or Portuguese. No geographic restrictions were applied.

### Information sources and search strategy

The strategy used keywords and controlled vocabularies, including MeSH terms, such as cancer/oncology patients, spirituality, spiritual well-being, spiritual care, spiritual interventions, and religion. Searches were performed by J.P.S.N on January 12th, 2025, across five electronic databases: PubMed, Embase, LILACS, Web of Science, and Scopus. Gray literature was searched via Proquest Dissertation and Theses, The Brazilian Digital Library of Theses and Dissertations, and Google Scholar. The complete search strategies are provided in Supplementary Table 1.

### Selection of source of evidence

Search results were imported into EndNote X9® (Thomson Reuters, Philadelphia, PA, USA) and Rayyan® (Cambridge, MA, USA). After the duplicate removal process, evidence selection was performed in two phases. In phase 1, two independent reviewers (J.P.S.N and C.M.P) screened titles and abstracts. In phase 2, the same reviewers assessed full texts to identify relevant studies. Full texts were retrieved for potentially eligible studies. Disagreements were resolved by consensus or consulting a third reviewer (A.R.S.S).

### Data charting

Data extraction focuses on the PCC framework, and it was performed independently by two reviewers using a standardized spreadsheet (Microsoft Excel®). Extracted variables included study title, authors, year, design, country, patient population, definitions of spirituality, and identified domains.

### Data items

Spirituality is generally understood as a dimension encompassing meaning, transcendence, coping, and connectedness. The central aim of this study is to deepen the understanding of this concept within oncology. To achieve this, definitions of spirituality provided by the studies, whether cited from other sources or originally formulated by the authors, were collected for analysis. The domains were consolidated into two primary categories: religion, referring to institutional beliefs, traditions, and practices; and faith, referring to spiritual beliefs and practices not necessarily linked to formal religious affiliation. Sources referring to both categories were classified as combined for descriptive purposes.

### Synthesis of results

Findings were narratively synthesized using a multi-methodological approach that encompassed qualitative interpretation and quantitative description. To map the recurring patterns in the conceptualizations of spirituality, thematic analysis was employed following Braun and Clarke’s (2006) [20] framework with an inductive approach. Thematic analysis is a qualitative method that enables the identification, analysis, and reporting of patterns within data. This analysis involved a thorough reading of the included articles, followed by listing and a comprehensive reading of the concepts and generating initial codes in the form of keywords across the text items. Candidate themes were constructed through iterative discussion of conceptual proximity among codes, definitions, and recurring interpretive patterns. After the creation of themes, the concepts were read again to define the placement of concepts within each theme followed by reporting of interpretations. All steps were conducted collaboratively by two researchers (J.P.S.N. and C.M.P.), who engaged in ongoing reflexive dialogue throughout the analytic process, from data collection to final report.

A separate descriptive analysis was conducted to examine the distribution of the main domain categories (Religion and Faith), alongside an integrated analysis of themes and domains. In addition, a complementary content-analytic step was performed to quantify the number of spirituality definitions allocated to each theme [21]. After theme construction, definitions were arranged chronologically to describe temporal patterns in the conceptualization of spirituality. This step was used as a contextual extension of the narrative synthesis rather than as an independent analytic methodology.

## Results

### Selection of evidence source

**Fig. 1:**
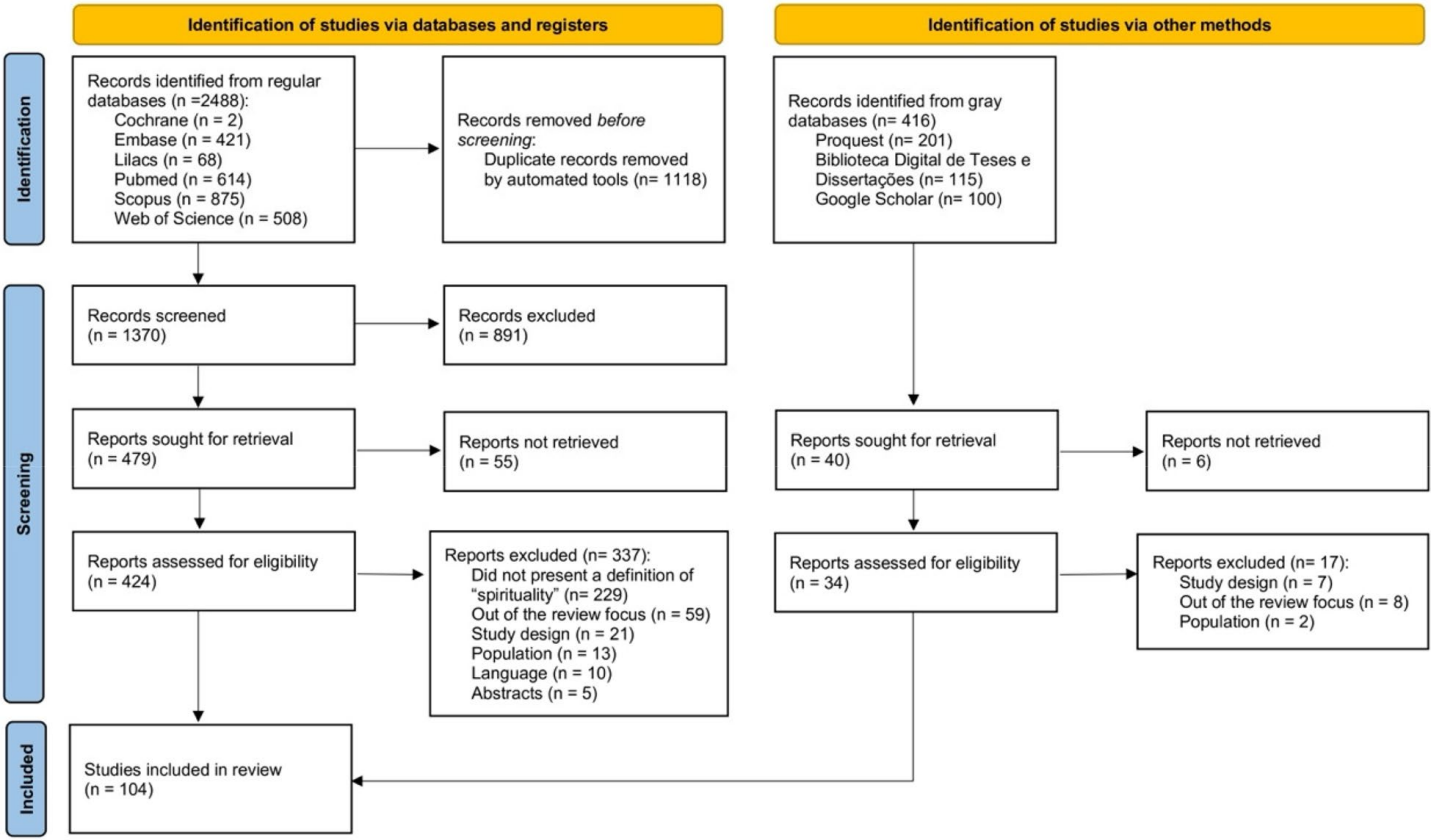
PRISMA flow diagram of literature search and study selection

A total of 2,488 records were identified from five electronic databases. After removing duplicates, 1,370 records were screened by title and abstract. 424 full-text were assessed with 87 included. In addition, 416 Gy literature records were retrieved from searches. Thirty-four records were assessed for eligibility, and 17 studies were included from gray literature. Overall, 104 studies met the eligibility criteria and were included in the synthesis (Fig. 1).

### Characteristics of included studies

The included studies spanned from 1987 to 2025 and covered diverse methodologies, including cross-sectional surveys (*n* = 61), qualitative descriptive studies (*n* = 30), randomized controlled trials (*n* = 5), and cohort designs (*n* = 8). Research originated from multiple regions, with the United States (*n* = 29), Brazil (*n* = 14), Turkey (*n* = 7), Iran (*n* = 6), The Netherlands (*n* = 6), and Italy (*n* = 6) most frequently represented.

### Results of individual sources

Across the included studies, spirituality in oncology was conceptualized in a variety of ways, highlighting its multidimensionality. Many studies portrayed spirituality as a resource that can enhance quality of life (*n* = 35) by fostering emotional well-being, comfort, and a sense of understanding. Others emphasized its function as a coping mechanism (*n* = 25) by supporting resilience, the search for meaning, and adaptation to the challenges faced in cancer treatment. Within the context of palliative care (*n* = 20), spirituality was often framed in terms of patients’ needs for existential support, reconciliation, and comfort at the end of life, informing individualized interventions and care planning. Additional studies examined the relationship between spirituality and psychological outcomes (*n* = 15), showing associations with reduced distress, anxiety, and depression, as well as with posttraumatic growth and emotional resilience. Across these perspectives, spirituality was generally distinguished from religiosity, though the two constructs were sometimes considered together.

### Synthesis of results

#### Thematic and content analysis

After reading and coding the 118 spirituality definitions extracted from all included studies, including peer-reviewed articles and gray literature, the authors constructed six themes through thematic analysis: (1) search for meaning and purpose; (2) transcendence and connection to something greater; (3) relational connectedness; (4) inner peace and well-being; (5) faith, belief, and the sacred; and (6) coping and existential resources. The following subsections present a general interpretation of the definitions encompassed within each theme. Counts (n) reported for each theme correspond to the number of spirituality definitions allocated to that theme and reflect a complementary content analysis (Supplementary Table 2).

#### Theme 1: search for meaning and purpose (*n* = 90)

This theme captures an exploration of the intrinsic dimensions of human experience, present in both religious and existential definitions of spirituality. For many cancer patients, the confrontation with illness and mortality generates existential disruption, leading to reflection on life, suffering, and personal values. In this context, spirituality is frequently understood as an active process of reconstructing meaning, closely linked to emotional resilience, hope, and the redefinition of values. Several studies highlighted that this search facilitates coping and supports psychological well-being. Its recurrence across different countries and cultural settings emphasizes its broad relevance across cultural and geographical contexts.

#### Theme 2: transcendence and connection to something greater (*n* = 56)

This theme captured a conceptually rich dimension of spirituality in the reviewed studies. It reflects the human inclination to move beyond material concerns and seek connection with a broader reality, often described as sacred, divine, universal, or transcendent. While earlier studies occasionally associated transcendence with the divine, more recent research has expanded this perspective to include experiences with nature, art, meditation, and personal reflection. Transcendence often intersects with other themes, particularly inner peace (Theme 4), as serenity is frequently depicted as an outcome of transcendent experiences. It is also closely connected to emotional resilience and spiritual growth, offering patients a pathway to reframe existential limitations.

#### Theme 3—relational connectedness (*n* = 50)

This theme encompassed relationships with oneself, others, the community, and, in some cases, nature and the divine. This perspective broadens the notion of spirituality, framing it as something shaped by interpersonal and social dynamics, while still remaining a personal dimension. Some studies emphasize that these ties go beyond family, extending to friends, caregivers, and others who provide presence and emotional support [22, 23]. What appears to matter most is the experience of connection and the formation of meaningful bonds, regardless of kinship. This theme also includes intrapersonal aspects, such as self-acceptance and inner reconciliation, which are often seen as central to spiritual well-being and are sometimes associated with the experience of inner peace. Furthermore, relational connectedness often intersects with other themes, particularly transcendence (Theme 2), suggesting that connection may function both as a pathway and as a goal within the spiritual journey.

#### Theme 4—inner peace and well-being (*n* = 12)

This theme captures definitions of spirituality centered on inner harmony, emotional balance, and existential comfort. The studies describe inner peace as a spiritual outcome recognized as a measurable aspect of spiritual well-being, especially in contexts such as palliative care and situations of emotional vulnerability. Practices such as meditation, prayer, personal reflection, and connection with nature are often mentioned as pathways to this state. Importantly, inner peace frequently overlaps with other themes, such as transcendence and relational connectedness, indicating that serenity tends to arise from a broader spiritual framework rather than as an isolated experience.

#### Theme 5 – faith, belief and the sacred (*n* = 66)

This theme reflects a traditional yet evolving dimension of spirituality, centered on faith in a higher power, reverence for the divine, and experiences of the sacred. Many studies describe spirituality through explicitly formal religious practices, such as prayer, devotion, and trust in God, while others emphasize more personal and intimate experiences of connection with the sacred that occur outside formal religious frameworks. Both perspectives can be seen in early and contemporary studies from diverse regions worldwide, indicating that the conceptualization of spirituality is shaped by regional and cultural contexts. This highlights the multidimensional nature of the concept of spirituality.

#### Theme 6—coping and existential resources (*n* = 18)

In this theme, spirituality is presented as an essential existential resource that helps patients cope with the challenges of cancer. It supports resilience, adaptation, and emotional regulation, playing a protective role in mitigating distress and reducing feelings of hopelessness and anxiety. Spirituality also contributes to existential well-being and overall quality of life, particularly during treatment periods marked by uncertainty and vulnerability. Notably, this theme closely interacts with meaning and purpose (Theme 1), highlighting how coping and existential resources are often mobilized through the reframing of personal values in the face of suffering.

### Temporal trends

**Fig. 2:**
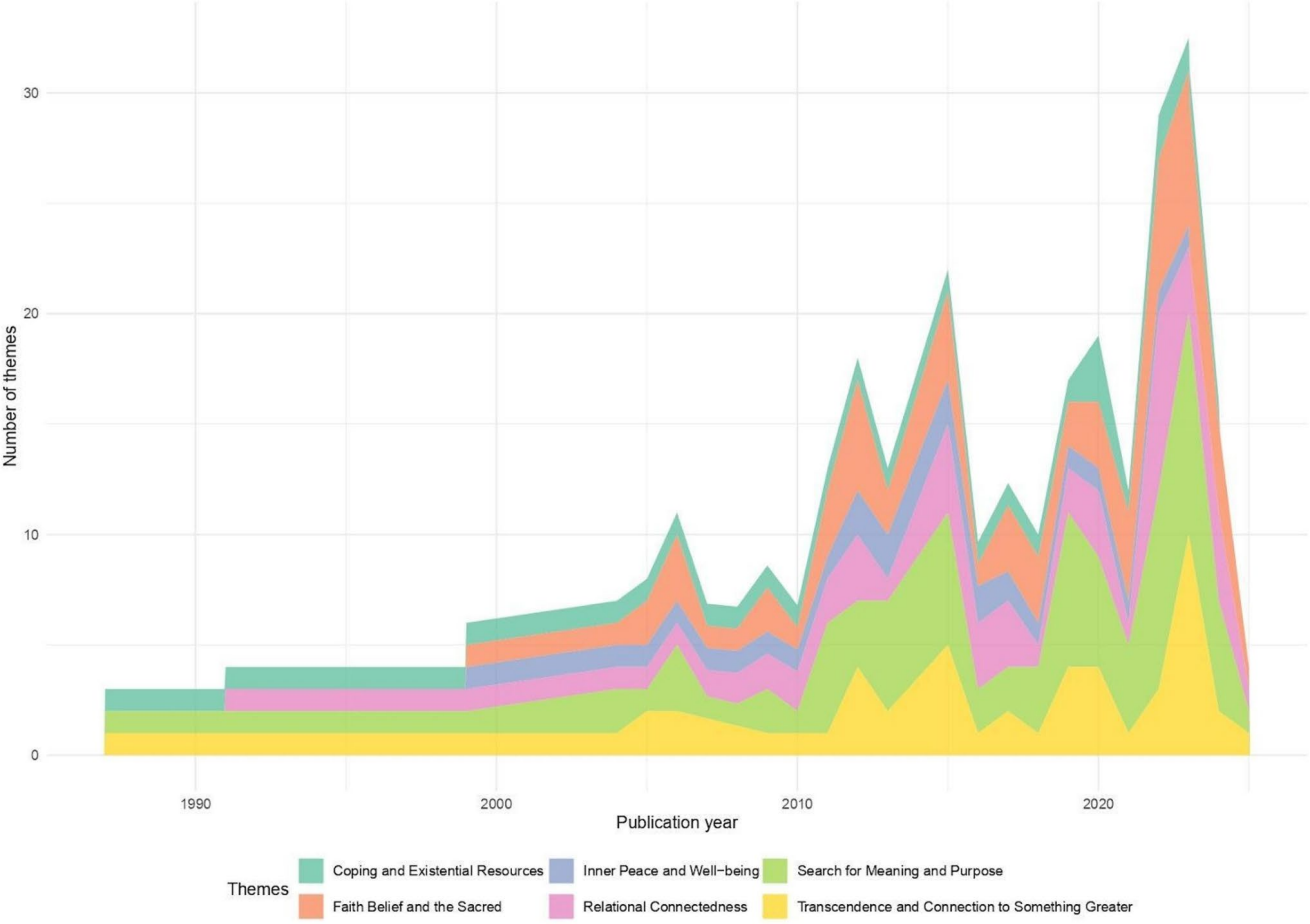
Stacked area chart illustrating the number of themes related to spirituality in analyzed publications

During the thematic analysis, we noticed a gradual shift in the way spirituality has been conceptualized over time, which led us to examine the performance of themes across different periods. The results show an ongoing evolution: earlier studies emphasized spirituality mainly through religious and traditional lenses, while more recent publications describe broader, more inclusive perspectives that highlight existential and relational aspects. This shift is particularly evident in the increased focus on themes such as search for meaning and purpose and relational connectedness in studies published after 2010. Research from the late 1990 s and early 2000 s often linked spirituality to transcendence through the sacred or divine, while more recent studies also recognize expressions of spirituality in nature, art, and personal reflection. This descriptive chronological assessment suggested a gradual shift in existential and psychosocial themes after 2015, alongside a relative decline in strictly religious definitions (Fig. 2).

### Domains

The domain analysis revealed a broad spectrum of spirituality-related aspects. Faith and religion were the most frequently cited domains, appearing in 26.9% and 63.5% of studies, respectively, with 20.2% referencing both. Religion was defined as a belief system involving institutional belonging, traditions, and practical commitments, whereas faith was defined as reliance on or adherence to spiritual beliefs and practices as a way of understanding the world. In this review, faith was used as a broader and less institutionally bound construct than religion.

**Fig. 3:**
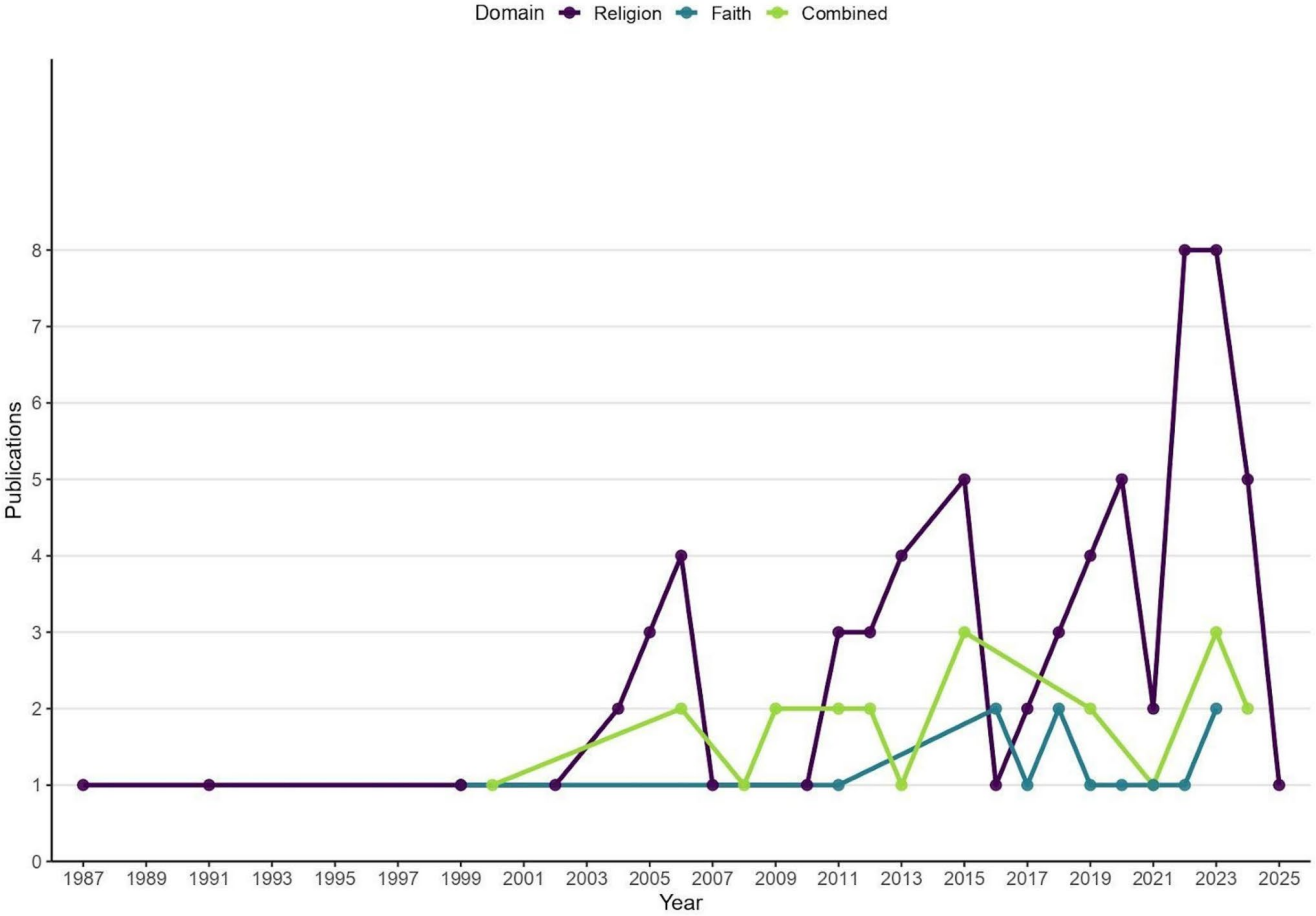
Line graph showing the number of publications per year across three domains: Religion (purple), Faith (blue), and Combined (green)

Temporal analysis revealed a general and consistent increase in the number of publications. Religion stands out as the most frequently cited domain, indicating its established presence and traditionality. Notably, since the early 2000 s, there has been a marked rise in studies that reference faith either independently or in conjunction with religion. This trend suggests a conceptual shift: faith is increasingly framed not solely as a part of the religious construct, but as a more individualized and experiential phenomenon, rooted in personal beliefs. The prominence of religion in the literature reflects its institutional and doctrinal foundations, whereas the growing attention to faith points to a diversification of spirituality (Fig. 3).

**Fig. 4:**
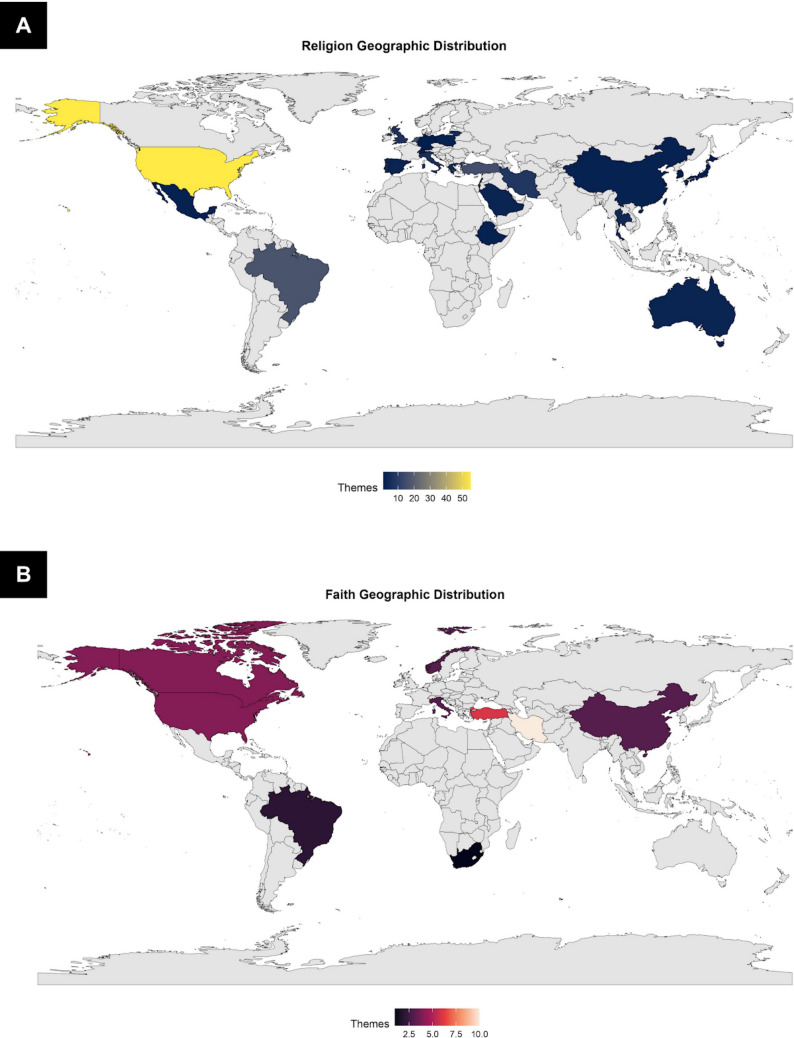
World maps with color gradient showing the geographic distribution and frequency of themes related to Religion (**A**) and Faith (**B**)

This distinction is further supported by the geographic analysis, which shows that religion-related studies are globally dispersed, showing its tradition, while faith-related research appears in more localized clusters, probably influenced by cultural and contextual factors. Religion-related themes are most prominent in countries such as the United States, Germany, Canada, and Australia and faith-related themes show concentrated presence in regions like Brazil, South Africa, and parts of Asia (Fig. 4). These patterns suggest that faith is present more strongly in culturally diverse settings where spiritual expression is shaped by local traditions and cultures.

### Integration of findings

**Fig. 5:**
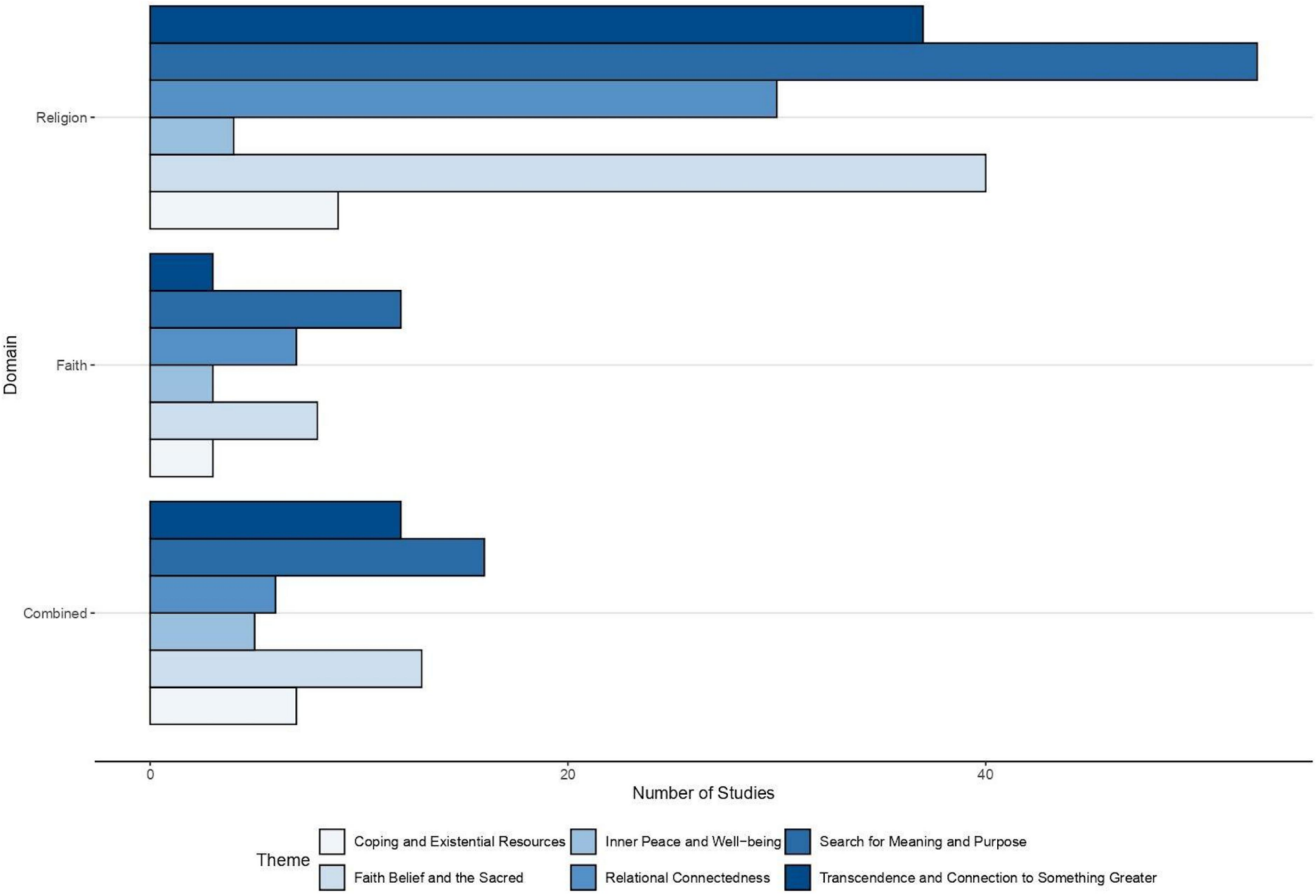
Horizontal bar chart comparing the number of studies across six themes within three domains: Religion, Faith, and Combined

The cross-analysis between themes and domains (Fig. 5) shows how the conceptualization of spirituality is wide, diverse, but interconnected. In the descriptive cross-analysis between themes and domains, the theme search for meaning and purpose was represented across all domains and accounted for the largest number of allocated definitions, supporting its central role in the conceptualization of spirituality in oncology.

Faith, belief and the sacred is the most dominant theme within the religion domain as expected, while inner peace and well-being and relational connectedness appear more frequently in the combined domain, suggesting that these dimensions are often experienced through both religious and existential pathways. Interestingly, transcendence and connection to something greater shows a balanced presence across domains, and coping and existential resources is also widely distributed, underscoring spirituality’s role as a psychological and emotional support mechanism in cancer care.

## Discussion

This scoping review provides a comprehensive overview of how spirituality has been conceptualized in oncology research over nearly four decades. Across our analysis, we noted that several authors and their writings have been influential in shaping the concepts and understandings of spirituality. Puchalski et al. (2009) [24] appears as a central reference across multiple themes, particularly in relation to meaning-making, transcendence, and faith. This work was frequently cited alongside Paloutzian and Ellison (1982) [25] and Peterman et al. (2002) [26], who developed the Functional Assessment of Chronic Illness Therapy–Spiritual Well-Being scale, which was used by several studies in this sample. Additionally, earlier works by Reed (1987) [27], Reed (1992) [28], Buchanan (1987) [29], Broten (1991) [30], and Frankl (1963) [31] provided important perspectives on transcendence, relationality, and belief in existential resilience.

The authors identified six core themes that define spirituality in the oncology literature, with search for meaning and purpose represented across the largest number of definitions in the complementary content analysis. The analysis also revealed a significant conceptual evolution, shifting from predominantly religious frameworks in earlier studies (1980s–2000s) to more existential perspectives in contemporary research. The geographical distinction between faith and religion may reflect cultural diversity and historical factors [32]. Time, culture, history, and geography were all important factors influencing the definition of spirituality, as observed in earlier studies within similar contexts [15, 16]. It is important to note that the term “faith”, identified as one of the domains in our analysis, may be interpreted differently depending on the reader’s cultural background. In this study, we adopted the meaning described by Peterman et al. (2002) [26] in the FACIT-Sp statement to classify the articles, that is, faith as a source of strength and comfort, and as the sense that “things will be okay”, derived from one’s spiritual beliefs. Accordingly, studies that associated this construct with their definition of spirituality were classified within the faith domain.

Previous research in this century has evaluated the literature to clarify the concept of spirituality. A review by De Brito Sena [16] employed a dimensional approach across all healthcare areas and interpreted some concepts similarly to the present study, such as the search for meaning and purpose, as well as certain forms of spiritual practices and interventions. That review also proposed a conceptual framework that closely aligns with the findings of the current study, offering valuable insights for interpreting the definition of spirituality. The present study advances that work by incorporating a temporal analysis to examine the evolution of the concept over time. Additionally, a review conducted by Balboni (2022) [17] provided further insight by focusing on health outcomes, finding promising results for the use of spirituality in improving patients’ quality of life and psychological well-being. Other studies within the nursing field highlighted the lack of knowledge among some healthcare professionals, as well as inconsistencies in terminology, which may hinder the application of important adjunctive tools in therapy [15]. Overall, the findings of the present review are consistent with recent evidence, supporting the central notion of spirituality as a multidimensional construct that encompasses meaning-making, transcendence, and connectedness, while extending beyond formal religious practices to include personal beliefs, interpersonal relationships, and existential exploration [33, 34].

Despite this extensive conceptual analysis, defining spirituality objectively within healthcare remains challenging. A notable effort toward consensus was made by Puchalski et al. (2014) [35], who proposed that spirituality is the aspect of humanity that refers to the way individuals seek and express meaning and purpose, and the way they experience their connectedness to the moment, to self, to others, to nature, and to the significant or sacred. Developed through an interprofessional consensus conference in the context of palliative care, this definition represents one of the most widely referenced attempts to standardize the concept across clinical settings and aligns closely with the multidimensional themes identified in the present review. This point was cited by articles in our sample and is explained by the complexity of the factors that influence its understanding [36]. The Physician Data Query® summary on spirituality in cancer care, developed by the National Cancer Institute (National Cancer Institute 2024) [37], emphasizes that there is no universally accepted definition of spirituality in healthcare, but provides relevant guidance on how spirituality may be understood and integrated into the care of patients with cancer. The World Health Organization (WHO) has not formally incorporated spirituality into its official health definition, despite repeated calls from member states since 1984 [38], primarily due to definitional ambiguity and medical practitioners’ demands for empirically measurable evidence that can be quantified as a “unique, uncontaminated construct”. However, the WHO has taken several initiatives to address this gap, including the development of the World Health Organization Quality of Life (WHOQOL) quality of life measure that attempts to capture spiritual dimensions cross-culturally, the incorporation of “spiritual wellbeing” terminology in the 2005 Bangkok Charter for Health Promotion [39], and most recently, the 2023 framework for achieving wellbeing that explicitly calls for “a positive vision of health that integrates physical, mental, psychological, emotional, spiritual and social wellbeing” [40].

This scoping review presents methodological and conceptual limitations that should be acknowledged. The restriction to studies published in English, Spanish, and Portuguese may have limited the cultural and geographical diversity of findings, potentially excluding important perspectives from other linguistic contexts. The exploratory nature of our objective precluded inferential quantitative analysis, limiting our ability to establish statistical relationships between variables or assess the strength of associations between conceptual domains. Additionally, the thematic analysis, while rigorous and transparent, involved interpretive judgment in categorizing definitions into themes, which may have influenced theme construction. The exclusive focus on oncology patients, although providing depth within this specific context, limits the generalizability of findings to other healthcare populations. Finally, as acknowledged throughout our analysis, the inherent difficulty in objectively defining spirituality in health remains a fundamental challenge that affects not only our study but the broader field, reflecting the complex nature of this construct.

This study examined the oncology literature to map and synthesize the concept of spirituality, revealing a wide diversity of definitions, perspectives, and temporal and cultural variations. Despite this conceptual richness, spirituality consistently appeared as a multidimensional construct, with “search for meaning and purpose” being the most prominent dimension. Given the recurring emphasis in the literature on both the diversity of information and the lack of professional knowledge about spirituality, these findings highlight the need for practical guidance. A global position statement from a major health organization, such as the WHO, or a leading medical society could help disseminate information to healthcare professionals about spirituality, outline how it can be addressed in clinical practice, and provide guidance on its practical application in patient care, supporting the development of standardized, culturally sensitive guidelines.

## Conclusion

This scoping review shows that spirituality in oncology is a multidimensional and context-dependent construct shaped by cultural, temporal, and contextual factors. Despite considerable conceptual diversity across studies, recurrent domains are consistently observed, suggesting a shared conceptual core articulated through different cultural and clinical frameworks. At the same time, the findings confirm that establishing a common or unified definition of spirituality remains elusive. By rigorously mapping definitions and conceptual domains, this review may contribute to greater conceptual clarity and provide an integrative overview that supports greater coherence in future research. Such mapping may also inform culturally sensitive approaches to spiritual care in oncology, helping to clarify how spirituality is conceptualized in relation to patient resilience and well-being, without imposing a single normative definition.

## Supplementary Information

Below is the link to the electronic supplementary material.

ESM 1 DOCX (14.1 KB)

ESM 2 DOCX (83.2 KB)

## Data Availability

All data supporting the conclusions of this study have been presented in the main text or Supplementary archives.
