# Supplementary material for: Mapping terminology and domains related to spirituality in oncology: scoping review
Source: Support Care Cancer. 2026 Jul 24;34(8):798. doi: 10.1007/s00520-026-11003-3 (PMC13400688; doi:10.1007/s00520-026-11003-3)
Supplement: Supplementary file 1 — DOCX (14.1 KB) [file 520_2026_11003_MOESM1_ESM.docx]

| **Source** | **Query (January 12th, 2025)** | **Results** |
| --- | --- | --- |
| **Databases** | | |
| PubMed | ("cancer patients"[All Fields] OR "oncology patients"[All Fields] OR "patients with cancer"[All Fields]) AND ("spirituality"[All Fields] OR "spiritual well-being"[All Fields] OR "spiritual care"[All Fields] OR "spiritual interventions"[All Fields] OR "spiritual assessment"[All Fields] OR "religion"[All Fields] OR "religiosity"[All Fields]) AND ("oncology"[All Fields]) | 614 |
| Scopus | TITLE-ABS-KEY ("cancer patients" OR "oncology patients" OR "patients with cancer") AND TITLE-ABS-KEY ("spirituality" OR "spiritual well-being" OR "spiritual care" OR "spiritual interventions" OR "spiritual assessment" OR "religion" OR "religiosity" ) AND TITLE-ABS-KEY ("oncology") | 875 |
| Embase | ('cancer patients' OR 'oncology patients' OR 'patients with cancer') AND ('spirituality'/exp OR 'spirituality' OR 'spiritual well-being'/exp OR 'spiritual well-being' OR 'spiritual care'/exp OR 'spiritual care' OR 'spiritual interventions' OR 'spiritual assessment' OR 'religion' OR 'religiosity') AND 'oncology'/exp | 421 |
| Cochrane Library | (”cancer patients" OR "oncology patients" OR "patients with cancer”) in Title Abstract Keyword AND (spirituality OR "spiritual well-being" OR "spiritual care" OR "spiritual interventions" OR "spiritual assessment" OR religion OR religiosity) in Title Abstract Keyword AND (oncology) in Title Abstract Keyword - (Word variations have been searched) | 2 |
| Web of Science | ((TS=(”cancer patients" OR "oncology patients" OR "patients with cancer”)) AND TS=(“spirituality" OR "spiritual well-being" OR "spiritual care" OR "spiritual interventions" OR "spiritual assessment" OR "religion" OR "religiosity")) AND TS=("oncology") | 508 |
| Lilacs | (("cancer patients" OR "oncology patients" OR "patients with cancer" OR "pacientes com câncer" OR "pacientes oncológicos")) AND (("spirituality" OR "espiritualidade" OR "espiritualidad" "spiritual well-being" OR "bem-estar espiritual" OR "bienestar espiritual" OR "cuidado espiritual" OR "spiritual care" OR "tratamento espiritual" OR "spiritual interventions" OR "spiritual assessment" OR "religion" OR "religião" OR "religion" OR "religiosity" OR "religiosidad" OR "religiosidade")) AND (("oncology" OR "oncologia")) | 68 |
| **Gray Literature** | | |
| Google Scholar | (”cancer patients" OR "oncology patients" OR "patients with cancer”) AND (“spirituality" OR "spiritual well-being" OR "spiritual care" OR "spiritual interventions" OR "spiritual assessment" OR "religion" OR "religiosity") AND ("oncology") | 100 |
| ProQuest | TI,AB(”cancer patients" OR "oncology patients" OR "patients with cancer”) AND TI,AB(“spirituality" OR "spiritual well-being" OR "spiritual care" OR "spiritual interventions" OR "spiritual assessment" OR "religion" OR "religiosity") AND TI,AB("oncology") | 201 |
| Biblioteca Digital Brasileira de Teses e Dissertações | (("cancer patients" OR "oncology patients" OR "patients with  cancer" OR "pacientes com câncer" OR "pacientes oncológicos"))  AND (("spirituality" OR "espiritualidade" OR "espiritualidad"  "spiritual well-being" OR "bem-estar espiritual" OR "bienestar  espiritual" OR "cuidado espiritual" OR "spiritual care" OR  "tratamento espiritual" OR "spiritual interventions" OR "spiritual assessment" OR "religion" OR "religião" OR "religion" OR "religiosity" OR "religiosidad" OR "religiosidade")) AND (("oncology" OR "oncologia")) | 115 |
