# Supplementary material for: Mapping terminology and domains related to spirituality in oncology: scoping review
Source: Support Care Cancer. 2026 Jul 24;34(8):798. doi: 10.1007/s00520-026-11003-3 (PMC13400688; doi:10.1007/s00520-026-11003-3)
Supplement: Supplementary file 2 — DOCX (83.2 KB) [file 520_2026_11003_MOESM2_ESM.docx]

| **Articles, Domains and Definitions** | | | | | | | | **Thematic Analysis**** | | | | | |
| --- | --- | --- | --- | --- | --- | --- | --- | --- | --- | --- | --- | --- | --- |
| **nº** | **Author** | **Title** | **Country** | **Year** | **Domains** | **Definitions of Spirituality** | **References** | **T1** | **T2** | **T3** | **T4** | **T5** | **T6** |
| **1** | Brady et al. | A case for including spirituality inequality of life measurement in oncology | United States of America | 1999 | Faith | "The way in which people understand and live their lives in view of their ultimate meaning and value" | Muldoon and King 1995 | X |  |  |  |  |  |
|  |  |  |  |  |  | "A present state of peace and harmony" | Hungelmann et al. 1985 |  |  |  | X |  |  |
|  |  |  |  |  |  | "The need for finding satisfactory answers to ultimate questions about the meaning of life, illness, and death" | Highfield and Cason 1983 | X |  |  |  |  | X |
|  |  |  |  |  |  | "Dealing with one’s adequacy to see the divinity in the status quo of life and then to rely on that transcendence to live above the present troublesome circumstances" | Brewer 1979 |  | X |  |  | X | X |
| **2** | Frick et al. | A clinical interview assessing cancer patients' spiritual needs and preferences | Germany | 2005 | Religion | "Combination of religious and existential (non-religious, related to meaning and purpose in life) well-being" | Paloutzian and Ellison 1982; Laubmeier et al. 2004 | X |  |  |  | X |  |
| **3** | Juškauskienė et al. | Spiritual Well-Being and Related Factors in Children With Cancer | Lithuania | 2023 | Combined | "An intrinsic aspect of humanity through which persons seek ultimate meaning, purpose, connectivity, and transcendence." | Weathers et al. 2016 | X | X |  |  |  |  |
| **4** | Wang et al. | Spiritual care needs and their attributes among Chinese in patients with advanced breast cancer based on the Kano model: a descriptive cross-sectional study | People's Republic of China | 2024 | Combined | "Means of experiencing themselves, the present, other people, the ultimate, and the natural environment" | Weathers 2021 |  |  | X |  |  |  |
| **5** | Bacoanu et al. | Spiritual Care for Cancer Patients at the End-of-Life | Romania | 2024 | Combined | "The need to find meaning, purpose and fulfilment in life, suffering and death. The need for hope and the desire to live. The need for faith and belief in oneself, in others and a power beyond oneself or God as defined by the individual” | Renetzky 1979, as cited on Batstone et al. 2020 | X |  | X |  | X | X |
| **6** | Abdalrahim et al. | Spiritual well-being among patients newly diagnosed with cancer in Jordan: thematic analysis. | Jordan | 2023 | Religion | "Is a trait that individuals seek meaning, purpose, and connection to a higher power that direct them to efficiently function toward desired life goals and wishes" | Lee 2019 | X | X |  |  |  |  |
| **7** | Majda et al. | Influence of Spirituality and Religiosity of Cancer Patients on Their Quality of Life | Poland | 2022 | Religion | "Is a dynamic and internal aspect of humanity through which people seek ultimate meaning, purpose and transcendence and experience relationships with themselves, family, others, community, society, nature and signifier or saint." | Ross 2006 | X | X | X |  | X |  |
| **8** | Ata et al. | Correlation of spiritual well-being with hope and depression in oncology patients: The case of Turkey | Turkey | 2021 | Faith | "Finding health and/or inner peace and feeling good through nonphysical means, such as searching for the meaning and purpose of life, praying, and meditation" | Jimenez-Fonseca et al. 2018 | X |  |  | X | X |  |
| **9** | Cengiz et al. | Effect of mindfulness-based therapy on spiritual well-being in breast cancer patients: a randomized controlled study | Turkey | 2023 | Religion | "Dynamic and intrinsic dimension of human life that relates to the way persons experience, express, and/or seek meaning, purpose, and transcendence, and the way they connect to the moment, to self, to others, to nature, and to the significant and/or the sacred" | Yilmaz et al. 2020 | X | X | X |  | X |  |
| **10** | Kestenbaum et al. | Spiritual AIM: assessment and documentation of spiritual needs in patients with cancer. | United States of America | 2022 | Religion | "Encompassing the dimension of life that reflects the need to seek meaning and direction, find self-worth and belonging to a community, and to love and be loved, facilitated by seeking reconciliation in broken relationships" | Shields et al. 2015 | X |  | X |  |  |  |
|  |  |  |  |  |  | "The aspect of humanity that refers to the way individuals seek and express meaning and purpose and the way they experience their connectedness to the moment, to self, to others, to nature, and to the significant or sacred." | Puchalski et al. 2009 | X |  | X |  | X |  |
| **11** | Yavuz and Koç | Religious attitude, spirituality and mental adjustment in Turkish geriatric oncology patients | Turkey | 2023 | Religion | "The feelings, thoughts, experiences and behaviors that usually arise from the search for “the sacred” without being connected to any religious organization." | Cirhinlioğlu 2014 |  |  |  | X |  |  |
| **12** | Cheng et al. | Improving spiritual well-being among cancer patients: implications for clinical care | People's Republic of China | 2019 | Combined | "A universal trait by which individuals look for hope and meaning in their life." | Smith 2017 | X |  |  |  |  | X |
| **13** | Biji et al. | Linguistic Validation of the Functional Assessment of Chronic Illness Therapy-Spiritual-Well-being-Expanded Version 4 Tool into Malayalam Language and its Feasibility in Advanced Cancer Patients Receiving Palliative Care | India | 2024 | Religion | "The way people find meaning and purpose in life, and how they experience connectedness to self, others, the significant or sacred." | Puchalski et al. 2009 | X |  | X |  | X |  |
| **14** | Sun et al. | Palliative care and spiritual well-being in lung cancer patients and family caregivers | United States of America | 2016 | Faith | "A connection with a larger reality that provides meaning to life and can be experienced through private and public devotions or through meditation, nature, or art" | Peteet 2013 |  | X | X |  | X |  |
|  |  |  |  |  |  | "aspect of humanity that refers to the way individuals seek and express meaning and purpose and the way they experience their connectedness to the moment, to self, to others, to nature, and to the significant or sacred." | NCP 2011 | X |  | X |  | X |  |
| **15** | Davis et al. | Changes in spiritual well-being and psychological outcomes in ovarian cancer survivors | United States of America | 2018 | Faith | "The construct of spirituality has been conceptualized as comprised of three components: meaning, peace, and faith." | Canada 2008 | X |  |  | X | X |  |
| **16** | Borelli et al. | Gratitude among advanced cancer patients and their caregivers: The role of early palliative care | Italy | 2022 | Faith | "The way people find meaning and purpose in the world and how they perceive their connection to self, others, the significant, or sacred." | Puchalski 2009 | X |  | X |  | X |  |
|  |  |  |  |  |  | “An intrinsic private relationship with a divine and spiritual transcendence.” | Loi 2021 |  | X |  |  | X |  |
| **17** | Moosavi et al. | Consequences of Spiritual Care for Cancer Patients and Oncology Nurses: a Qualitative Study | Iran | 2019 | Religion | "The essence of being and an aspect of being human. This dimension refers to the way a person searches for meaning and purpose in life, as well as the relationship between himself and others, nature, and the sacred." | Puchalski 2009 | X |  | X |  | X |  |
|  |  |  |  |  |  | "A person's search for meaning and purpose in life." | Tanyi 2002 | X |  |  |  |  |  |
| **18** | Rabitti et al. | The assessment of spiritual well-being in cancer patients with advanced disease: which are its meaningful dimensions? | Italy | 2020 | Faith | “A context in which people can make sense of their lives, and feel whole, hopeful and peaceful even in the midst of life’s most serious challenges” | Brady 1999 | X |  |  | X |  | X |
|  |  |  |  |  |  | “One’s striving for and experience of a connection with the essence of life of which the experiences of meaning in life and connectedness are central elements” | Visser 2010 | X | X |  |  |  |  |
| **19** | Rong et al. | Spirituality as a Mediator Between Social Support and Benefit Finding Among Advanced Cancer Patients | People's Republic of China | 2023 | Religion | "A supernatural belief system that is beyond the material world" | Lindeman 2012 |  | X |  |  | X |  |
|  |  |  |  |  |  | "The notion that people strive to make sense of, and derive meaning from, life events and seek to connect with the self, others, and their community, underpinned by a personal belief system." | Timmins 2017 | X |  | X |  | X |  |
| **20** | Almeida Filho et al. | Spirituality in the uncertainty of illness: the perspective of oncology patients | Brazil | 2023 | Religion | "One of the dimensions of the human experience. It is expressed by the inner search of human beings and by the constructed meaning, through their beliefs, values, and principles, which can rescue the meaning of life and, thus, enable interrelationships with the divine, with nature and with oneself." | Siqueira et al. 2017 | X | X | X |  | X |  |
| **21** | Garssen et al. | How Spirituality Helps Cancer Patients with the Adjustment to their Disease | The Netherlands | 2015 | Religion | "The propensity to make meaning through a sense of relatedness to dimensions that transcend the self in such a way that it empowers and does not devalue the individual. This relatedness can be experienced intrapersonally (as a connectedness within oneself), interpersonally (in the context of others and the natural environment), and transpersonally (referring to a sense of relatedness to the unseen, God, or power greater than the self and ordinary source)." | Reed 1992 | X | X | X |  |  |  |
| **22** | Almaraz et al. | Religiosity, Emotions and Health: The Role of Trust/Mistrust in God in People Affected by Cancer | Spain | 2022 | Religion | “Spirituality is a way of being in the world in which a person feels a sense of connectedness to self, others, and/or a higher power or nature; a sense of meaning in life; and transcendence beyond self, everyday living, and suffering” | Weathers et al. 2015 | X | X | X |  |  | X |
| **23** | Phenwan et al. | The Meaning of Spirituality and Spiritual Well-Being among Thai Breast Cancer Patients: A Qualitative Study | Thailand | 2019 | Combined | "The essence of a human being: The meaning of life, feeling of connectedness to the transcendental phenomena such as the universe or god" | Wang and Lin 2016; Bai and Lazenby 2015; Zamaniyan et al. 2016 | X | X |  |  |  |  |
| **24** | Heuschkel et al. | Spirituality in Oncology: Relations between Spirituality, Its Facets, and Psychological and Demographic Factors in Cancer Patients in Germany | Germany | 2024 | Religion | "Values or beliefs as well as a personal quest for purpose and meaning in life. This is independent from individual religious beliefs." | Buck et al. 2009; Tanyi 2002 | X |  |  |  |  |  |
| **25** | Feng et al. | Exploring the relationship between spiritual well-being and death anxiety in patients with gynecological cancer: a cross-section study | People's Republic of China | 2021 | Religion | "The way individuals seek and express meaning and purpose in life and how they experience their connectedness to the moment, to self, to others, to nature, and the significant or sacred" | Puchalski et al. 2009 | X |  | X |  | X |  |
| **26** | Aktaş and Uğur | The effect of physical and psychological symptoms on spiritual well-being and emotional distress in inpatient cancer patients | Turkey | 2023 | Faith | "Individual’s effort to express himself, to interpret his relationship with other people, nature or power he holds to be sacred, to seek purpose and embrace it": | Delgado-Guay et al. 2011 | X |  | X |  | X |  |
| **27** | Yılmaz et al. | Determination of Comfort Levels and Spiritual Care Needs of Gynecologic Cancer Patients with Abdominal Drains: A Cross-Sectional Descriptive Study in Turkey | Turkey | 2024 | Religion | "The essence of the person, the search for meaning and purpose in life." | Meneguin et al. 2020 | X |  |  |  |  |  |
| **28** | Mazzotti et al. | Predictors of existential and religious well-being among cancer patients | Italy | 2011 | Religion | "The way in which people understand their lives in view of their ultimate meaning and value" | Koenig et al. 2001 | X |  |  |  |  |  |
|  |  |  |  |  |  | "The personal search for purpose and meaning in life as well as connection to a sacred or transcendent reality" | Hill et al. 2000; Puchalski 2002 | X | X |  |  | X |  |
| **29** | Yang et al. | Mediating role of spirituality on the relationships between posttraumatic stress and posttraumatic growth among patients with cancer: A cross-sectional study | Taiwan | 2023 | Faith | "The process and result of fulfilling the meaning of life." | Hsiao et al. 2013 | X |  |  |  |  |  |
| **30** | Ratshikana-Moloko et al. | Spiritual Care, Pain Reduction, and Preferred Place of Death Among Advanced Cancer Patients in Soweto, South Africa | South Africa | 2020 | Religion | "The way people find meaning and purpose during traumatic, illness and life-threatening events" | Puchalski et al. 2019 | X |  |  |  | X | X |
| **31** | Hamdan et al. | Religious Belief and Social Support Among Cancer Patients in Saudi Arabia | Saudi Arabia | 2020 | Religion | "An attachment to religious values or a matter of spirit, meaning it is a state of connecting oneself to God, nature, one another, and the deepest parts of ourselves." | Balboni et al. 2007; Moadel et al. 1999 |  | X | X |  | X |  |
| **32** | Schultz et al. | Distinguishing Between Spiritual Distress, General Distress, Spiritual Well-Being, and Spiritual Pain Among Cancer Patients During Oncology Treatment | Israel | 2017 | Faith | "A dynamic and intrinsic aspect of humanity through which persons seek ultimate meaning, purpose, and transcendence, and experience relationship to self, family, others, community, society, nature, and the significant or sacred." | Puchalski et al. 2014 | X | X | X |  | X |  |
| **33** | Arefian et al. | Proposing and evaluating a model of depression, stress, resilience and spirituality in relation to pain in women with breast cancer: Investigating the mediating role of mindfulness | Iran | 2023 | Religion | "The search for answers to existential questions about the meaning of life and one's relationship with the sacred or transcendent" | Toivonen et al. 2018 | X | X |  |  | X |  |
| **34** | Esmaelli et al. | Exploring the religious and spiritual coping experience of cases via cancer: A qualitative research | Iran | 2015 | Religion | "One’s ability to seek purpose and meaning, to make the connection, and to pursue a transcendental value" | Yong et al. 2007 | X | X |  |  |  |  |
| **35** | Nixon and Narayanasamy | The spiritual needs of neuro-oncology patients from patients' perspective | United Kingdom | 2010 | Religion | "The non-physical part of our life which is considered to be the essence of our being. It gives meaning and purpose to our existence. Some associate it with religion, while others do not." | Narayanasamy 2007; Swinton 2001; DOH 2003; SEHD 2002; Ross 1997 | X |  |  |  | X |  |
| **36** | Mesquita et al. | The use of religious/spiritual coping among patients with cancer undergoing chemotherapy treatment | Brazil | 2013 | Religion | "A person's essence, a search for meaning and purpose in life" | Como 2007 | X |  |  |  |  |  |
| **37** | Levy and Chan | Spiritual coaching in cancer patients: treating the spirit as well as the disease | United States of America | 2006 | Religion | "A spiritual state or things affecting the spirit" | Puchalski and Romer 2000 |  |  |  |  |  |  |
|  |  |  |  |  |  | "That which allows a person to experience transcendent meaning in life" | Puchalski and Romer 2000 | X | X |  |  |  |  |
| **38** | Buck and Meghani | Spiritual expressions of African Americans and Whites in cancer pain | United States of America | 2012 | Religion | "Three concentric domains of coexisting connectedness. The human being experiences first personal integration (connection to self), then integration with others (connection to friends, family, all humans), and finally integration with transcendent dimensions (connection to nature and/or a Supreme Being)." | Buck 2006 |  | X | X |  | X |  |
|  |  |  |  |  |  | "An inherently human experience, sometimes involving religious structures, that connects the person to others, nature, and/or a Supreme Being providing meaning and purpose" | Buck 2006 | X | X | X |  | X |  |
| **39** | Chaar et al. | Evaluating the impact of spirituality on the quality of life, anxiety, and depression among patients with cancer: an observational transversal study | Lebanon | 2018 | Faith | "A framework that provides people with a sense of ultimate purpose and meaning in life, offering people stability, support, and direction in critical times" | Hill and Pargament 2003 | X |  |  |  | X | X |
| **40** | Rohde et al. | Associations between sex, age and spiritual well-being scores on the EORTC QLQ-SWB32 for patients receiving palliative care for cancer: A further analysis of data from an international validation study | Norway | 2019 | Religion | "A broad concept with room for many perspectives and no universally agreed definition. In general, it includes a sense of connection to something greater than ourselves, and it typically involves a search for meaning in life" | Cobb, Puchalski, and Rumbold 2012 | X | X |  |  |  |  |
|  |  |  |  |  |  | "The search for meaning in one`s life and the living of one`s life on the basis of one`s understanding of that meaning. It may involve some or all of the following: having or finding (a) sustaining relationship with self and others; (b) meaning beyond one`s self; (c) meaning beyond immediate events; and (d) explanation for events and/or experiences" | Vivat 2008 | X | X | X |  |  | X |
| **41** | Bovero at al. | The Spirituality in End-of-Life Cancer Patients, in Relation to Anxiety, Depression, Coping Strategies and the Daily Spiritual Experiences: A Cross-Sectional Study | Italy | 2019 | Religion | "The feeling stemming from the person’s connection with the transcendent dimension of the existence, i.e., the meaning or the sense of purpose" | Peterman et al. 2002 | X | X |  |  |  |  |
| **42** | Cipriano-Steffens et al. | "Let Go, Let God": A Qualitative Study Exploring Cancer Patients' Spirituality and Its Place in the Medical Setting | United States of America | 2020 | Religion | "The transcendent, addressing ultimate questions about life’s meaning" | Idler et al. 2003 | X | X |  |  |  |  |
| **43** | Sprik et al. | Using patient-reported religious/spiritual concerns to identify patients who accept chaplain interventions in an outpatient oncology setting | United States of America | 2018 | Religion | "A process, a search for the sacred" | Pargament 2013 |  |  |  | X |  |  |
| **44** | Visser, Meezenbroek and Garssen | Does spirituality reduce the impact of somatic symptoms on distress in cancer patients? Cross-sectional and longitudinal findings | The Netherlands | 2018 | Religion | "One's striving for and experience of connectedness with the essence of life" | Jager Meezenbroek et al. 2012 |  |  | X |  |  |  |
| **45** | Balboni et al. | Religiousness and spiritual support among advanced cancer patients and associations with end-of-life treatment preferences and quality of life | United States of America | 2007 | Religion | "The search for ultimate meaning through religion or other paths" | National Cancer Institute. Spirituality in cancer care. http://www.nci.nih.gov/cancertopics/pdq/supportivecare/spirituality/ | X |  |  |  | X |  |
| **46** | Renz, Schütt Mao and Cerny | Spirituality, psychotherapy and music in palliative cancer care: research projects in psycho-oncology at an oncology center in Switzerland | Switzerland | 2005 | Religion | "Energetic reality adding impetus and creativity to healing and dying beyond any medically possible assessment" | Not Referenced |  | X |  |  | X | X |
| **47** | Kruizinga et al. (A) | Images of God and attitudes towards death in relation to spiritual wellbeing: an exploratory side study of the EORTC QLQ-SWB32 validation study in palliative cancer patients | The Netherlands | 2017 | Religion | "The aspect of humanity that refers to the way individuals seek and express meaning and purpose and the way they experience their connectedness to the moment, to self, to others, to nature, and to the significant or sacred" | Puchalski et al. 2009 | X |  | X |  | X |  |
| **48** | Kruizinga et al. (B) | An assisted structured reflection on life events and life goals in advanced cancer patients: Outcomes of a randomized controlled trial (Life InSight Application (LISA) study) | The Netherlands | 2019 | Faith | "The process of seeking and expressing meaning and purpose in life and the experience of connectedness to others, the self, nature, the moment, and a higher being" | Puchalski et al. 2009 | X | X | X |  |  |  |
| **49** | Musarezaie et al. | Investigation of the SWB and its relation with demographic parameters in patients with breast cancer referred to an oncology hospital affiliated to the Isfahan university of medical sciences | Iran | 2013 | Religion | "The way individuals seek and express meaning and purpose, and the way they experience their connectedness to the moment, to self, to others, to nature, and to the significant or sacred" | Puchalski et al. 2009 | X |  | X |  | X |  |
|  |  |  |  |  |  | "Connecting with our higher selves and with God and the Universal Spirit, the Creator, the Life Force, or whatever name you desire to give to that Divine energy" | Surbone and Baider 2010 |  | X | X |  | X |  |
| **50** | Purnell, Andersen and Wilmot JP | Practice and Spirituality in the Psychological Adjustment of Survivors of Breast Cancer | United States of America | 2009 | Combined | "A broader search for meaning, purpose, and value in life and the former is a means by which these may be found" | Frame 2003; Frame et al. 2005; Helminiak 2001 | X |  |  |  | X |  |
|  |  |  |  |  |  | "Awareness of a transcendent dimension" | Elkins et al. 1998 |  | X |  |  |  |  |
|  |  |  |  |  |  | "Innate capacity and tendency to move towards knowledge, love, meaning, hope, transcendence, connectedness and compassion" | Fukuyama and Sevig 1999 | X | X | X |  |  | X |
|  |  |  |  |  |  | "The personal search for purpose and meaning in life as well as connection to a sacred or transcendent reality" | Hill et al. 2000 | X | X |  |  | X |  |
| **51** | Ben-Arye et al. | Is a biopsychosocial-spiritual approach relevant to cancer treatment? A study of patients and oncology staff members on issues of complementary medicine and spirituality | Israel | 2006 | Religion | "A sense of meaning and purpose in life, faith, and comfort with existential concerns." | McClain et al. 2003 | X |  |  |  | X |  |
|  |  |  |  |  | Religion | “Discuss spiritual aspects (meaning of disease, life, and death and making a mindful decision in choosing treatment).” | Not Referenced | X |  |  |  |  | X |
| **52** | Laubmeier, Zakowski SG and Bair | The role of spirituality in the psychological adjustment to cancer: a test of the transactional model of stress and coping | United States of America | 2004 | Religion | "The ability of individuals to find meaning in their life (e.g., finding a life purpose or a sense of life fulfillment and satisfaction)" | Frankl et al. 1963 | X |  |  |  |  |  |
|  |  |  |  |  |  | "A combination of religious well-being (harmony with God or a higher power) and existential well-being (nonreligious sense of meaning and purpose in life)" | Paloutzian and Ellison 1982 | X | X |  |  | X |  |
| **53** | Rohani et al. | Health-related quality of life and the predictive role of sense of coherence, spirituality and religious coping in a sample of Iranian women with breast cancer: a prospective study with comparative design | Iran | 2015 | Religion | "A form of self-transcendence, and defines it as an individualized awareness of one’s inner self and a sense of conjunction with a powerful dimension or purpose" | Reed 1987 | X | X | X |  |  |  |
| **54** | Whitford and Olver | The multidimensionality of spiritual wellbeing: peace, meaning, and faith and their association with quality of life and coping in oncology | Australia | 2012 | Religion | "A personal search for meaning and purpose in life, connection with a transcendent dimension of existence, and the experiences and feelings associated with that search" | Peterman et al. 2002 | X | X |  |  | X |  |
| **55** | Otis-Green et al. | An integrated psychosocial-spiritual model for cancer pain management | United States of America | 2002 | Religion | "Interconnectedness with others, nature, and the Transcendent." | Dossey and Halifax 2001 |  | X | X |  |  |  |
| **56** | Mattos | Religious/spiritual coping and quality of life of patients with hematologic cancer undergoing hematopoietic stem cell transplantation. | Brazil | 2021 | Religion | "Related to transcendence and faith, it is found across all cultures and ages, representing a way for individuals to find meaning and connection with the self, with others, or with the sacred. It is considered a broad term, as religiosity is associated with specific religions." (Translated from Portuguese – Brazil) | Puchalski et al. 2018 | X | X | X |  | X |  |
| **57** | Silva et al. | Spiritual dimension of pain and suffering control of advanced cancer patient | Brazil | 2015 | Combined | "A quality of individuals whose interior life is oriented toward God, mysticism or what is sacred, which goes beyond science and instituted religion." | Saporetti 2008 |  | X |  |  | X |  |
| **58** | Vaz et al. | Assessment of the level of spirituality and hope in cancer patients. | Brazil | 2022 | Religion | "It is a phenomenon interconnected with traditional roots and culture, merging body and mind, and providing meaning, strength, and faith throughout the cancer journey." (Translated from Portuguese – Brazil) | Gifford et al. 2019 | X |  |  |  | X | X |
| **59** | Huang et al. | Effects of symptoms and complementary and alternative medicine use on the yang deficiency pattern among breast cancer patients receiving chemotherapy | Taiwan | 2015 | Combined | "An awareness of something greater than an individual's power. It is often expressed through religion or prayer, or both" | American Cancer Society. Complementary and alternative  medicine. <http://www.cancer.org/treatment/treatmentsandsideeffects/complementaryandalternativemedicine/index> |  | X |  |  | X |  |
| **60** | Freire, Vasconcelos and Silva | Spiritual and religious assistance to cancer patients in the hospital context | Brazil | 2017 | Religion | "A characteristic of the individual, which can include belief in a God, and establish a spiritual connection of being with the cosmos and with other people." | Geronasso and Coelho 2012; Fornazari and Ferreira 2010 |  | X | X |  | X |  |
| **61** | Köktürk Dalcali and Kaya | Spiritual Care Needs of Patients in Oncology Units and Nursing Practices in Turkey: A Qualitative Study | Turkey | 2022 | Religion | "The aspect of humanity that refers to the way individuals seek and express meaning and purpose, and the way they experience their connectedness to the moment, to self, to others, to nature and to the significant or sacred" | Puchalski et al. 2009 | X |  | X |  | X |  |
| **62** | Ebenau et al. | Spiritual care by nurses in curative oncology: a mixed-method study on patients' perspectives and experiences | The Netherlands | 2020 | Religion | "The dynamic dimension of human life that relates to the way persons (individual and community) experience, express and/or seek meaning, purpose and transcendence, and the way they connect to the moment, to self, to others, to nature, to the significant and/or the sacred. The spiritual field is multidimensional: (i) existential challenges; (ii) value-based considerations and attitudes; and (iii) religious considerations and foundations" | Agora Studygroup Guideline Spiritual Care, Spiritual Care: National Guideline, Version: 1.0 - 2010 | X | X | X |  | X |  |
| **63** | Krupski et al. | Spirituality influences health related quality of life in men with prostate cancer | United States of America | 2006 | Religion | "The ultimate meaning and value of life" | Muldoon and King 1995 | X |  |  |  |  |  |
|  |  |  |  |  |  | "The search for peace and harmony" | Hungelmann et al. 1996 |  |  | X |  |  |  |
| **64** | Noguchi et al. | Reliability and validity of the Functional Assessment of Chronic Illness Therapy-Spiritual (FACIT-Sp) for Japanese patients with cancer | Japan | 2004 | Religion | Those aspects of human life relating to experiences that transcend sensory phenomena. This concept is not equivalent to religion, though for many people the spiritual dimension of their lives includes a religious component. The spiritual aspects of human life may be viewed as an integrating component, binding together the physical, psychological, and social components. Spirituality is often perceived, in clinical practice, as being concerned with meaning and purpose and can be a more immediate issue for those nearing the end of life | WHO 1983 | X | X |  |  | X | X |
| **65** | Ahmadi et al. | Spiritual coping strategies: Relationship with physical, anxiety, and depression symptoms of patients with cancer | Iran | 2023 | Religion | "Dimension of human being and show the relationship between the person and cosmos. Integration makes the human being hopeful and transcends him beyond time, place and material interests" | Salajegheh 2014 |  | X |  |  | X |  |
| **66** | Ascencio-Huertas and Flores-Gonzáles | Spiritual well-being and coping in palliative care oncology patients | Mexico | 2025 | Religion | "Spirituality is the dimension of human life that relates to the ways in which individuals/communities express and/or seek meaning, purpose, and transcendence, and how they connect with the present moment, with themselves, with others, with nature, with the meaningful, and/or with the sacred." | Nolan et al. 2011 | X | X | X |  | X |  |
| **67** | Assimakopoulos et al. | Religiosity and its relation to quality of life in Christian Orthodox cancer patients undergoing chemotherapy. | Greece | 2009 | Combined | "A personal search for meaning and purpose in life, a connection with a transcendent dimension of existence and the experiences and feelings associated with that search" | Peterman et al. 2002 | X | X |  |  | X |  |
| **68** | Batista et al. | Conception of spirituality of cancer patients undergoing  antineoplastic treatment | Brazil | 2021 | Combined | "The personal quest to understand the meaning and finitude of life, as well as the awareness, or belief, that there is something sacred in the world that transcends the limits of religious rituals" | Koenig et al. 2012 | X |  |  |  | X | X |
| **69** | Ben-Arye et al. | Integrating complementary medicine and supportive care: patients' perspectives toward complementary medicine and spirituality | Israel | 2012 | Religion | "Interest in a spiritual (e.g., the meaning of life and its purpose) or religious quest. | Not Referenced | X |  |  |  | X |  |
|  |  |  |  |  |  | "The way in which people understand their life in view of their ultimate meaning and value" | Muldoon and King 1995 | X |  |  |  |  |  |
|  |  |  |  |  |  | “A sense of meaning and purpose in life, faith, and comfort with existential concerns” | McClain et al. 2003 | X |  |  |  | X |  |
| **70** | Cannon et al. | Interplay between spirituality and religiosity on the physical and mental well-being of cancer survivors | United States of America | 2022 | Religion | “An individual’s sense of peace, purpose, connection to others, and beliefs about the meaning of life” | National Cancer Institute 2012. http://www.cancer.gov/cancertopics/pdq/supportivecare/spirituality/Patient/page1. | X |  | X | X | X |  |
| **71** | Cook and Silverman | Effects of music therapy on spirituality with patients on a medical oncology/hematology unit: A mixed-methods approach | United States of America | 2013 | Combined | "Search for meaning and purpose in life and attempt to promote virtues such as love, wisdom, and truth" | Chappel and McKee 1992 | X |  |  |  | X |  |
|  |  |  |  |  | Combined | "Relationships with the universe, people, and the self" | Walker 1995 |  | X | X |  |  |  |
|  |  |  |  |  | Combined | "The aspect of humanity that refers to the way individuals seek and express meaning and purpose and the way they experience their connectedness to the moment, to self, to others, to nature, and to the significant or sacred" | Puchalski et al. 2009 | X |  | X |  | X |  |
| **72** | Gudenkauf et al. | Spirituality and Emotional Distress Among Lung Cancer Survivors | United States of America | 2019 | Religion | "Connection to something greater than oneself through which humans pursue a sense of purpose, meaning, and peace" | Puchalski et al. 2014 | X | X |  | X |  |  |
| **73** | Heshmati et al. | The effects of mindfulness-based stress reduction on spiritual well-being and hope in patients with breast cancer: A randomized controlled trial | United Kingdom | 2024 | Religion | "A subjective experience of or connection to something beyond the self, such as the transcendent or divine" | Pargament 2011 |  | X |  |  | X |  |
| **74** | Holt et al. | Religiosity and physical and emotional functioning among African American and White colorectal and lung cancer patients | United States of America | 2011 | Religion | "Search for meaning and purpose in life" | Thoresen 1998 | X |  |  |  |  |  |
| **75** | Kang et al. | Addressing the religious and spiritual needs of dying patients by healthcare staff in Korea: patient perspectives in a multi-religious Asian country | Republic of Korea | 2011 | Religion | "The search for ultimate meaning through religion or other paths" | National Cancer Institute: Spirituality in cancer care. www.nci.nih.gov/cancertopics/pdq/spportivecare/spirituality/ | X |  |  |  | X |  |
| **76** | Kebede et al. | Spiritual well-being and associated factor among adult cancer patients in Hawassa University Comprehensive Specialized Hospital, Oncology Center, Hawassa, Ethiopia | Ethiopia | 2024 | Religion | "A sense of connection with people, having meaning and purpose in life, and, additionally, believing in and relating to a superior or higher force" | Jafari et al. 2010 | X | X | X |  | X |  |
| **77** | Kırca et al. | Evaluation of the Spiritual Care Needs of Patients with Cancer in a Chemotherapy Unit in Turkey | Turkey | 2023 | Combined | "An aspect of one's life related to the search for meaning, purpose, and connection with a higher power and enables the individual to function effectively toward life purpose" | Lee 2019; National Cancer Institute 2015 | X | X |  |  |  |  |
| **78** | Macciò et al. | Survival as a clinical outcome and its spiritual significance in a cohort of patients with advanced central pelvic neoplastic disease undergoing total pelvic evisceration: a poorly debated issue | Italy | 2023 | Religion | "A search for the ultimate meaning of existence through religion or other paths, including the faith in supernatural being or powers" | King et al. 1995 | X | X |  |  | X |  |
|  |  |  |  |  |  | "The set of aspects of experience, not necessarily linked only to the commitment to religious practices but, in general, directed toward the search for a global sense of peace, meaning, purpose, and connection" | Supportive and Palliative Care Editorial Board 2022; Magnano et al. 2019 | X | X |  | X |  |  |
|  |  |  |  |  |  | "The aspect of humanity that refers to the way individuals seek and express meaning and purpose and the way they experience their connectedness to the moment, to self, to others, to nature, and to the significant or sacred" | Puchalski et al. 2009 | X |  | X |  | X |  |
| **79** | Nash and Nash | Reflections on Using Metaphors in Exploring Spiritual and Religious Needs with Young People with Cancer and their Families | United Kingdom | 2015 | Religion | "The aspect of humanity that refers to the way individuals seek and express meaning and purpose, and the way they experience their connectedness to the moment, to self, to others, to nature and to the significant or sacred" | Puchalski and Ferrell 2010 | X |  | X |  | X |  |
| **80** | Lenneke et al. | Transcending the Suffering in Cancer: Impact of a Spiritual Life Review Intervention on Spiritual Re-Evaluation, Spiritual Growth and Psycho-Spiritual Wellbeing | The Netherlands | 2020 | Religion | "The relationship with the self, with others, nature, the world, a higher power, meaning or presence; Transcendence has to do with the ability to transcend one’s own suffering and to exceed one’s restrictions and limitations; Finally, meaning and meaning making refer to the manner in which people give meaning to, or find meaning in life." | Puchalski et al. 2009; Weathers et al. 2016 | X | X | X |  |  | X |
| **81** | Riklikiene et al. | Translation and Validation of Spiritual Well-Being Questionnaire SHALOM in Lithuanian Language, Culture and Health Care Practice | Lithuania | 2018 | Religion | "An expression, framework, source of, or searching for, transcendent meaning to life" | Ferrell et al. 2003; Puchalski et al. 2009 | X | X |  |  | X |  |
|  |  |  |  |  |  | "Immediate experience or reactions to life" | Lazenby 2010 |  |  | X |  |  |  |
|  |  |  |  |  |  | "The way people “experience their connectedness to the moment, to self, to others, to nature, and to the significant or sacred” | Puchalski et al. 2009 | X |  | X |  | X |  |
| **82** | Rippentrop et al. | The Relationship of Religiosity and Spirituality to Quality of Life Among Cancer Patients. Journal of Clinical Psychology in Medical Settings | United States of America | 2006 | Combined | "A person's unique search, which may or may not be as a member of an organized religion, for what is sacred in life" | Larson, Swyers, and McCullough 1997 |  |  |  | X |  |  |
| **83** | Silva et al. | Filling gaps in experiences religious understanding of people living with cancer in palliative care: a phenomenological qualitative study | Brazil | 2023 | Combined | "The search to guide life, in line with this ultimate meaning of life or with the fundamental values or with something beyond oneself (transcendence)" | Pessini 2018; Safra 2018 | X | X |  |  |  |  |
| **84** | Mabena and Moodley | Spiritual Meanings of Illness in Patients with Cervical Cancer | South Africa | 2012 | Combined | "Consists of seven constructs: love, belonging, respect, divine, positivity, gratitude, hope, peace, meaning and purpose, morality and ethics, appreciation of beauty, resolution/death" | Galek et al. 2005 | X | X | X | X | X | X |
|  |  |  |  |  |  | "Developmental and conscious process, characterized by two movements of transcendence, either deep within the self or beyond the self" | Vachon, Fillion and Achille 2009 |  | X | X |  |  |  |
| **85** | Vallurupalli et al. | The role of spirituality and religious coping in the quality of life of patients with advanced cancer receiving palliative radiation therapy | United States of America | 2011 | Combined | “An individual’s sense of peace, purpose, and connection to others, and beliefs about the meaning of life” | National Cancer Institute 2009 | X |  | X | X | X |  |
| **86** | Vespa et al. | ‌Evaluation of intrapsychic factors, coping styles, and spirituality of patients affected by tumors | Italy | 2011 | Faith | "A source of faith, hope, trust, and meaning, and in the context of interpersonal, transpersonal, and/or intrapersonal (intrapsychic) processes and experiences" | Emavardhana and Tori 1997; Woods and Ironson 1999 | X |  | X |  | X | X |
| **87** | Zernicke et al. | The eCALM Trial: eTherapy for Cancer Applying Mindfulness. Exploratory Analyses of the Associations Between Online Mindfulness-Based Cancer Recovery Participation and Changes in Mood, Stress Symptoms, Mindfulness, Posttraumatic Growth, and Spirituality | Canada | 2016 | Faith | "Experiences and emotions associated with the search for value and meaning in life, increasing purpose and connection" | Peterman et al. 2002; Lechner et al. 2008; Visser et al. 2010 | X |  | X |  |  |  |
| **88** | Trovo | When "a word of affection comforts more than any medicine": needs and expectations of the patients under palliative care. | Brazil | 2006 | Combined | "The set of an individual's core essence as a person, their relationship with an infinite being and with others, and their pursuit of fulfillment, meaning, and purpose in life." | McEwen 2005 | X | X | X |  | X |  |
| **89** | Borman | Spirituality and religiosity and their relationship to the quality of life in oncology patients | United States of America | 1999 | Religion | "An inner state of being, or the heart or force within a person." | Beck 1986; Goldsmith 1992 |  |  |  | X |  |  |
|  |  |  |  |  |  | "That which enables the growth of positive and creative values in the human being." | Booth 1984 |  | X |  |  | X |  |
|  |  |  |  |  |  | "Not just an absence of pathology, but as a clear sense of meaning or purpose in life" | Miller 1997 | X |  |  | X |  |  |
|  |  |  |  |  |  | "Encompasses humans' need to find satisfactory answers to questions about the meaning of life, illness, and death." | Ellerhorst-Ryan 1988 | X |  |  |  |  | X |
|  |  |  |  |  |  | "Transcendent dimension outside of material existence, one that can definitely be experienced, but never bounded, controlled, or possessed." | Miller 1997 |  | X |  |  |  |  |
| **90** | Broten | Spiritual care given by nurses and spiritual well-being of terminally ill cancer patients | United States of America | 1991 | Religion | "A quality that goes beyond religious affiliation, that strives for inspiration, reverence, awe, meaning and purpose, even in those who do not believe in God." | Murray and Zentner 1985 | X | X |  |  | X |  |
|  |  |  |  |  |  | "The need for finding answers to questions about the meaning of life, illness and death." | Highfield and Cason 1983 | X |  |  |  |  | X |
|  |  |  |  |  |  | "Integration of the whole person in harmony with God and relationships with other people leading to meaning and purpose, love and relatedness, and forgiveness." | Shelley and Fish 1988 | X |  | X |  | X |  |
|  |  |  |  |  |  | "The human capacity for values and beliefs which transcend the material, physical world which provides personal meaning and integrates all other aspects of life." | Williams 1985 | X | X |  |  | X |  |
| **91** | Buchanan | Loneliness and spiritual well-being of hospitalized and healthy adults: a quality of life study | United States of America | 1987 | Religion | "The dimension of human beings within which the spirit, the invariable self and the unifying force of life, searches for meaning and satisfaction in life's events and relates to a higher being through religious experiences." | Colliton 1981 | X | X |  |  | X |  |
| **92** | Burt | Hope and spirituality and their relationship to the overall quality of life in cancer patients | United States of America | 2011 | Combined | "An immaterial aspect of human  nature that never dies." | Meraviglia 2004 |  | X |  |  |  |  |
|  |  |  |  |  |  | "An inner state of being  or the force within an individual" | Goldsmith 1992 |  |  |  | X |  |  |
|  |  |  |  |  |  | "The expressions and experiences of an individual’s spirit in unique and dynamic practice reflecting faith in a Supreme Being, integration of the dimensions of mind, body, and spirit and connectedness with self, others, nature, or God" | Meraviglia 1999 |  | X | X | X | X |  |
|  |  |  |  |  |  | "A clear sense of meaning or purpose in life and incorporates finding satisfactory answers to questions concerning illness, death, and the meaning of life." | Miller 1997; Ellerhorst-Ryan 1988 | X |  |  |  |  | X |
|  |  |  |  |  |  | "Transcendent dimension outside of material existence, one that can definitely be experienced, but never bound, controlled, or possessed" | Miller 1997 |  | X |  |  |  |  |
| **93** | Chequini | Spirituality and religiosity in patients with head and neck cancer: a cross-sectional study during the COVID-19 pandemic | Brazil | 2022 | Religion | "A dynamic and intrinsic aspect of humanity through which people seek meaning, purpose, and transcendence, and experience relationships with self, family, others, community, society, nature, and the significant or sacred" | Puchalski et al. 2014 | X | X | X |  | X |  |
|  |  |  |  |  |  | "An individual and subjective dimension, broader than religiosity, which may or may not involve belief in God or adherence to a religion." | Koenig 2012 | X |  |  |  | X |  |
| **94** | Pereira | Experiences of Young People with Oncological Illness: The Meaning of Religiousness and Spirituality in Coping Processes and Strategies | Portugal | 2022 | Religion | "A dimension that provides individuals with well-being, inner peace, and an appreciation for the gratuitousness, meaning, and significance of human relationships." | Pinto 2011 | X |  | X | X |  |  |
|  |  |  |  |  |  | "An intrinsic dimension of the human being, particularly when one professes or, even indirectly, experiences a religion. It encompasses matters such as the meaning and purpose of life." | Not Referenced | X |  |  |  | X |  |
| **95** | Fuller | Spirituality and quality of life in breast cancer patients undergoing radiation treatment: A multifactor analysis | United States of America | 2006 | Religion | “An attachment to values of the spirit...the  immaterial aspect of a person that never dies” | Meraviglia 2004 |  | X |  |  | X |  |
|  |  |  |  |  |  | "Spirituality is concerned with universal issues of purpose and meaning of life and is the part of the human essence that strives for transcendental values. Spirituality is characterized by the capacity to seek purpose and meaning, to have faith, to love and forgive, to worship, and to see beyond present circumstances, and enables a person to rise above or transcend suffering." | Rousseau 2000 | X | X | X |  | X | X |
| **96** | Houk | Providing pastoral care to oncology patients: Effective techniques | United States of America | 2005 | Religion | "A commitment to choose, as the primary context for understanding and acting, one’s relatedness with all that is." | Neville and Kirkwood 1995 |  | X | X |  |  |  |
| **97** | Kantor | Factors Influencing Psychological Distress in Patients with Cancer | United States of America | 2013 | Religion | "Defined within transcendence, value guidance, or religiosity, and provides meaning and purpose for individuals" | Coyle 2002 | X | X |  |  | X |  |
| **98** | Laubmeier | The role of spirituality in the psychological adjustment to cancer | United States of America | 2000 | Combined | "The direct, personal experience of God, the awareness of a higher power that operates in all aspects of existence." | Ellison 1983 |  | X |  |  | X |  |
|  |  |  |  |  |  | "The search for meaning and purpose in life, for life’s ultimate significance." | Reed 1992 | X |  |  |  | X |  |
|  |  |  |  |  |  | "A combination of religious well-being (harmony with God) and existential well-being (non-religious sense of meaning and purpose in life)." | Paloutzian and Ellison 1982 | X |  |  | X | X |  |
| **99** | Lo | Do daily spiritual experiences buffer the effects of stoicism and fatalism on psychological distress and treatment seeking in a Chinese American cancer sample with pain? | United States of America | 2012 | Combined | "Incorporates personal beliefs in a power apart from one’s own existence and is a state of being harmonious or sharing interconnectedness with oneself, others, nature, and the transcendent." | Chiu 2001; Lukkahatai 2004 |  | X | X | X | X |  |
| **100** | Lourenção | Stress and spirituality in patients diagnosed with lung cancer | Brazil | 2015 | Combined | "Everything that gives meaning to life, independent of religion, and provides the ability to endure feelings that generate guilt, anger, and anxiety, fostering positive energies and improving people's quality of life." | Guerrero et al. 2011 | X |  |  | X |  | X |
| **101** | Naves | Quality of life and subjective well-being assessment in oncology: A study with bone cancer survivors | Brazil | 2013 | Combined | "The “central axis” in an individual’s life, enhanced by the experience of illness." | Liberato and Macieira 2008 | X |  |  |  |  | X |
| **102** | Radl | The Effects of Self-Book© Art Therapy on Emotional Distress in Female Cancer Patients: A Randomized Controlled Trial | United States of America | 2015 | Religion | "An individual’s sense of peace, purpose, and connection to others, and beliefs about the meaning of life." | National Institutes of Health. 2013. http://www.nlm.nih.gov/medlineplus/cancer.h | X |  | X | X | X |  |
| **103** | dos Reis | Association between psychosocial well-being, religiosity, and spirituality in patients with visible sequelae from oncologic surgery in the head and neck region. | Brazil | 2016 | Religion | "That which gives meaning to life, a personal feeling that fosters interest in others and in oneself, capable of helping to endure debilitating feelings of guilt, anger, and anxiety, and is a concept that may or may not involve religion." | Saad et al. 2001 | X |  | X |  |  | X |
|  |  |  |  |  |  | "An experience of contact with a dimension that goes beyond the realities considered tangible in human life, transcending them, and closely connected to the supernatural and the mystical." | Koenig 2012 |  | X |  |  | X |  |
|  |  |  |  |  |  | "A personal search for meaning and purpose in life, a connection with a transcendent dimension of existence, and the experiences and feelings associated with that search and connection." | Büssing et al. 2014; Zinnbauer et al. 1999 | X | X |  |  |  |  |
| **104** | Vasconcelos | The devotion to Our Lady in oncologic patients | Brazil | 2008 | Combined | "The search for the meaning of existence, which does not imply that a person already possesses faith in a sacred being or the transcendent, nor that they are necessarily inclined to adhere to a religious system." | Valle 1998; Giovanetti 2005 | X |  |  |  | X |  |
|  |  |  |  |  |  | "A subjective experience in which the human being undertakes a deep inward journey, seeking to experience reality as a whole, with which they feel integrated." | Giovanetti 2005 | X | X | X |  |  |  |
|  |  |  |  |  |  | "An oceanic feeling, that is, dynamic, vital, and creative, which may or may not be related to institutionalized religions." | Ancona-Lopez 2005 | X | X |  | X | X |  |

*<http://www.cancer.org/treatment/treatmentsandsideeffects/complementaryandalternativemedicine/index>

**Theme 1: Search for Meaning and Purpose; Theme 2: Transcendence and Connection to Something Greater; Theme 3: Relational Connectedness; Theme 4: Inner Peace and Well-being; Theme 5: Faith Belief and the Sacred; Theme 6: Coping and Existential Resources
